# Flexible use of quorum and numerosity principles in evaluation of social and non-social cues in group contexts

**DOI:** 10.1186/s41235-025-00703-9

**Published:** 2026-01-12

**Authors:** Jessica Savoie, Francesca Capozzi, Jelena Ristic

**Affiliations:** 1 Department of Psychology, McGill University, 2001 Avenue McGill College, Montreal, QC H3A 1G1 Canada; 2 Department of Psychology, University of Quebec in Montreal (UQAM), Montreal, QC Canada

**Keywords:** Multi-agent, Group perception, Gaze, Quorum-like evaluation

## Abstract

Although gaze following is an important socio-interactive process, little is known about how this behavior is affected when multiple gaze cues are encountered in groups. Emerging research suggests that both visual consistency of cues and group size may play a role. For example, in groups of three, a minority of target-congruent gaze cues (or 1/3 faces looking at the target) have been found to facilitate target responses, whereas in groups of five, a majority of target-congruent gaze cues (or 3/5 faces looking at the target) were needed for the same effect. Here, in two preregistered experiments, we provide a high-powered conceptual replication of these past experiments and extend them to examine the possible uniqueness of responses to gaze using a comparison with arrows. We found that a minority of target-congruent gaze and arrow cues significantly facilitated target responses regardless of group size. Furthermore, we found that additional target-congruent cues, either gaze or arrows, led to further significant response facilitation. Thus, initially, responses were facilitated by a minority proportion of target-congruent cues with response times continuing to decrease with increasing numerosity of cues’ spatial consistency toward the target. This suggests that humans may use both quorum-like and numerosity evaluation flexibly to guide responses in contexts presenting with multiple social or non-social cues.

**Supplementary Information:**

The online version contains supplementary material available at 10.1186/s41235-025-00703-9.

## Significance Statement

Imagine approaching a pedestrian intersection to cross the street. Likely, multiple individuals are also waiting to cross, and monitoring for different environmental cues, including what other individuals are doing, the pattern of traffic, and signalization. While humans frequently navigate complex environments like this, little remains known about how we interpret and act upon multiple social and non-social visual cues present in such contexts. Here we provide new insight into how people perceive and respond to multiple directional cues, such as social gaze and non-social arrows. By testing responses cued by various proportion of groups’ consistent social or non-social cues, we show that a minority of target-congruent cues significantly facilitated responses (engaging quorum-like evaluation). We also show that each additional target-aligned cue produced further response gains (engaging numerosity-based evaluation). These findings reveal that people flexibly combine quorum-like and numerosity-based evaluation strategies when processing complex and diverging visual information in group contexts. In everyday life, such mechanisms may help to rapidly interpret the intentions of others or the focus of attention within crowds, classrooms, teams, or other social settings where multiple cues are simultaneously present. Beyond social contexts, this research shows that similar cognitive principles may govern how we respond to non-social directional signals, such as arrows or signs in cluttered visual environments.

**Supplementary Information:**

The online version contains supplementary material available at 10.1186/s41235-025-00703-9.

## Introduction

Human gaze provides a rich source of social information. Evolutionarily, information from gaze promoted survival by signaling a food source or an approaching predator (Emery, 2000; Langton et al., 2000). Indeed, human infants as young as two days old show high interest in eye gaze (Farroni et al., 2002, 2004) and reliably start to follow the gaze direction of others by four months of age (Farroni et al., 2000; Hood et al., 1998), with this behavior persisting throughout adulthood (Capozzi & Ristic, 2018; Frischen et al., 2007; Sato et al., 2007). While most research has thus far assessed how humans perceive, process, and respond to gaze from a single person, in real life, we often encounter multiple individuals who can display consistent or inconsistent gaze by looking in the same or different directions (Ristic & Capozzi, 2022). Cognitive research on these multi-agent contexts remains scarce, although a burgeoning literature on social and interaction perception is starting to indicate that social groups may elicit preferential cognitive and neural processing due to social and/or perceptual salience (Colombatto et al., 2024; Papeo et al., 2017; Vestner et al., 2020; Yan et al., 2024). However, the question of how the consistency in group gaze direction influences observers’ behavior remains rarely addressed and deserves further investigation (Capozzi et al., 2018; Ma et al., 2024; Sun et al., 2017). In this study, we investigated how the consistency of gaze and comparison arrow cues (as a test of the social uniqueness) within groups of three and five affected observers target responses.

Past research shows that gaze cues are perceived differently depending on the size of the social group. Well-established research on the effects of one person gaze cues shows that humans spontaneously follow where others are looking and are significantly faster to respond to targets appearing at the gazed-at (vs. gazed-away) locations (Driver et al., 1999; Friesen & Kingstone, 1998). However, it remains unclear how multiple inconsistent or consistent gaze cues in a group would affect gaze following and influence responses to gazed-at targets. As discussed by Ristic and Capozzi (2022), in groups of less than five, social information tends to be extracted from individual group members, and thus, a single person’s target-congruent gaze can significantly facilitate responses. In line with this, Capozzi et al. (2018) presented participants with an image depicting a group of three or five faces that looked at a possible response target location with varying spatial consistency (i.e., one, two, three, four, or five faces looking toward the response target). The results indicated that for a group of three, one face looking at the target was sufficient to elicit faster responses to the gazed-at target (i.e., minority of consistent cues), while in a group of five, three out of five faces looking at the target (i.e., majority of consistent cues) were needed for the same effect to emerge. Additional research indicated that in crowds of ten or more, responses tend to be facilitated by the direction of the majority of target-congruent cues, which are presumed to involve ensemble perception to facilitate the extraction of the average crowd properties rather than individual cue evaluation (Baek & Chong, 2020; Whitney & Leib, 2018). Supporting this idea, using a group size of ten, Sun et al. (2017) found that a majority of target-consistent gaze cues (i.e., 6/10) were needed to produce faster target responses.

Taken together, these results suggest that the number of target-congruent gaze cues needed to facilitate responses is influenced by both spatial visual cue consistency, such as the number of individuals looking in the same direction as well as factors involved in group perception, such as group size. In line with this, Capozzi et al. (2018) proposed that facilitated response behavior to multiple gaze cues in humans may be driven by a threshold of congruent gaze cues similar to a quorum (or a relevant proportion, but not necessarily a majority) in which the response facilitation threshold fluctuates with group size to ultimately converge with a majority congruency representation as group size increases to a crowd (Ristic & Capozzi, 2022). This parallels quorum-sensing communication mechanisms found across diverse animals species, including single-cell organisms, ants, fish, and other group-living animals (Cornforth et al., 2014; Franks et al., 2015; Ward et al., 2008, 2012) in which a likelihood of an individual animal performing a behavior is found to increase once a threshold proportion of other group members performing the same behavior is met (Ward et al., 2008, 2012). Quorum-sensing behavior is thought to promote survival, group decision-making and cohesiveness (Seeley et al., 1991).

In the present study, we aimed to replicate the initial findings by Capozzi et al. (2018) in a high-powered design and to additionally examine whether behavior guided by quorum-like evaluation in humans would be unique to biological cues like gaze. Such a comparison is crucial for delineating the mechanisms of more complex human social behaviors and how they may relate comparatively to similar processes reported in other animal species. Some studies have suggested that the proportion-based judgment necessary in group cognition reflects the recruitment of domain-general processes driven by cues’ directional information rather than biological or behavioral information (for discussion, see Flavell et al., 2022). Thus, the comparison with control stimuli such as arrows is crucial for elucidating the mechanisms underlying group cognition.

Here in two experiments, we examined how different proportions of target-consistent biological gaze and comparison directional arrows presented in group contexts affected target responses. If humans utilized quorum-like evaluation, facilitated target responses could emerge even with a minority of target-consistent cues. If quorum-like evaluation was specific to social cues like gaze, we expected to find differences across gaze and arrow cues. Finally, based on Capozzi et al. (2018), we expected that the cue-target congruency threshold for responding may vary with group size (i.e., between groups of three and five).

## Experiment 1

Experiment 1 presented groups of three faces or three arrows to examine how changes between zero, one, two, or three target-congruent cues influenced target response speed. By manipulating cue type between biological gaze and directional arrow, Experiment 1 also examined whether the responses observed for gaze cues were different from responses observed for arrow cues. If humans used quorum-like evaluation, we expected to find target-facilitated responses with a minority of target-congruent gaze cues. If such behavior was specific to sociobiological cues, like gaze, we expected that the data for gaze and arrow cues would diverge. The study was preregistered at https://osf.io/tqky3.

## Methods

### Participants

A sample size of 151 was estimated a priori using GPower (Faul et al., 2007) as required to detect the smallest theoretically relevant effect magnitude reflecting the difference between target-congruent and target-incongruent response times (RTs) for each numerosity adjacent target-congruent cue as reported by Capozzi et al. (2018) [(1- β) =.80, *⍺* = 0.05, *dz* =.213] and as estimated from pilot data. Hundred and sixty-six volunteer participants were recruited (Women = 70%, men = 30%, other = 1.19%, age = 26.17 ± 6.69) from the student participant pool (SONA, N = 82) or from Prolific Academic (https://www.prolific.com/, *N* = 85). They received course credit or monetary payment (£6.00/hour), respectively. Data from 157 participants were analyzed. Based on preregistration criteria, data were excluded from analyses if overall response accuracy was lower than chance (50%) and/or if after erroneous trial exclusions (RT > 1200 ms or RT < 200 ms) fewer than 70% of trials remained. All participants reported native English proficiency, normal or corrected to normal vision, no history of psychiatric illness, not taking any attention altering medication, and no history of head injury resulting in loss of consciousness. Informed consent was obtained from all participants by way of electronic signature. All methods and procedures were approved by McGill University’s Ethics Board.

### Apparatus and Stimuli

The experiment was implemented using the online platform Testable (www.Testable.org). The experiment launched on participants’ individual computers, with the size of the stimuli adjusted for each screen size, using the size calibration function available within Testable.

**Fig. 1:**
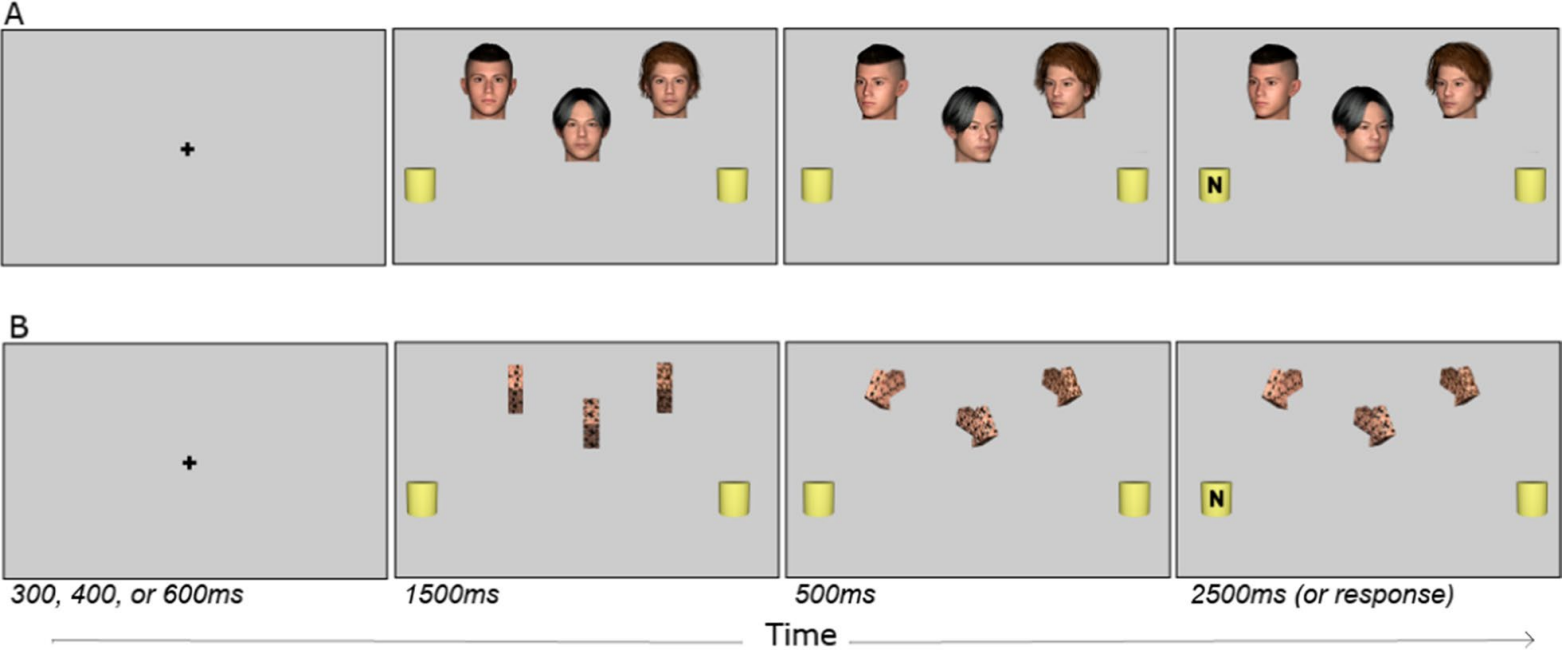
Example stimuli and procedure for gaze (**A**) and arrow (**B**) cues*.* Participants were first presented with a fixation cross for a randomly chosen interval of 300, 400, or 600 ms. This was followed by the presentation of three stimuli in a facing orientation. After 1500 ms, the cues turned to the left or right, to create one of the cue consistency conditions (zero, one, two, or three consistent cues). After 500 ms, the response target appeared on one of the two placeholders. The cues and the target remain visible for 2500 ms or until response. The stimuli are not drawn to scale

Figure 1 illustrates the stimuli. Stimuli were created using the DAZ3D studio (4.2.2) and edited using GIMP software (2.10.36). The stimuli included images of three human heads each depicting a different identity and measuring about 3.5 × 2.5 cm. Corresponding arrows (each measuring about 2x.6 cm when facing forward) were rendered as three-dimensional objects and colored using digital pixel sheets (30px pixel size) created by scrambling the image of one of the three face identities. Overall, groups measured 12 × 5.5 cm (faces) and 10.5 × 3.5 cm (arrows).

Response targets were black capital “N” and “H” letters (.6 x.8 cm, Sans Serif font) and appeared on one of the three-dimensional yellow cylinders (2 x.2.1 cm) positioned on the left or right side of fixation at an eccentricity of approximately 9 cm. The black central fixation cross measured.5 cm. All stimuli were rendered in color and set against a uniform gray (#d6d6d6) background.

The cues were positioned at 300 or 200 pixels above fixation. The response targets were positioned at the horizontal meridian. The distance between each cue was constant at 250 pixels as measured from the center of each cue.

### Design

The experiment was a repeated measures design with four factors, *Cue type* (2: face, arrows), *Cue Consistency* (4: zero, one, two, three), *Target location* (2: left, right), and *Target type* (2: N, H). All variables were intermixed. Intermixed design was chosen to control for any effects of context that could arise with alternative was of trial presentations such as blocking (e.g., Zhang et al., 2023). *Cue type* varied between face and arrow cues, which were shown equally and equiprobably and were kept consistent within displays. *Cue consistency* denoted cue visual consistency between zero, one, two, or three cues indicating the same spatial location (left or right). *Target location* varied between left and right, such that on any given trial, a target could appear on the left or the right side of fixation with equal probability. Finally, *Target type* varied between the letters N and H equally. Importantly, and for the purpose of analyses, we created a composite *Cue-target congruency* variable which combined the factors of Cue consistency and Target location. As such, this composite variable yielded conditions in which zero, one, two, or three cues indicated the upcoming target location.

There was a total of 288 combinations of Cue type x Cue consistency x Target location x Target type combinations. Half of those combinations (144) presented gaze cues, while the other half presented arrow cues. Each Cue consistency condition (0, 1, 2, or 3 cues consistently cuing the target) was presented 72 times, with the target appearing on the left 36 times and on the right 36 times, with target “H” shown 18 times, and the target “N” showed 18 times in each left or right location. Each Cue type (gaze, arrow) appeared in each possible position 48 times. Thus, all variables varied equiprobably and presented no relevant spatial information about the type of cues, cue numerosity, target location, or target identity.

### Procedure

Participants accessed the study via a weblink. The study launched in full screen. Trials began with a presentation of a fixation cross in a random duration of 300, 400, or 600 ms. Next, either three faces or three arrows were shown facing forward, along with the two yellow peripheral target placeholders. After 1500 ms, each cue turned either to the left or right side in various spatial consistencies (i.e., 0, 1, 2, or 3 faces turned toward a side, and the remaining faces turned toward the opposite side). 500 ms later, a response target appeared in one of the two placeholders on the left or right. This stimulus onset delay time was chosen to align with past work which did not show modulation of the group cuing effects with cue-target time (Capozzi et al., 2018, 2021). The cues and the target display remained visible until a response was made or 2500 ms had elapsed. Participants were asked to identify the target quickly and accurately by pressing the “v” or “g” keys on the keyboard, with the target identity-response key assignment counterbalanced across participants. Participants were instructed that cues did not provide information about target location and to maintain a central fixation throughout.

The experiment consisted of 4 blocks of 72 trials, with 10 practice trials run at the start. Participants were given an opportunity to take breaks after each block and were reminded of their response keys. The experiment took about 25 min.

### Results

Overall response accuracy in target identification was 92.48%. Accuracy was not analyzed further. Response time anticipations (RT < 200 ms) and timed-out responses (RT > 1200 ms) accounted for 0.39% and 2.59% of trials, respectively. These trials were removed from participant files and were not analyzed.

**Fig. 2:**
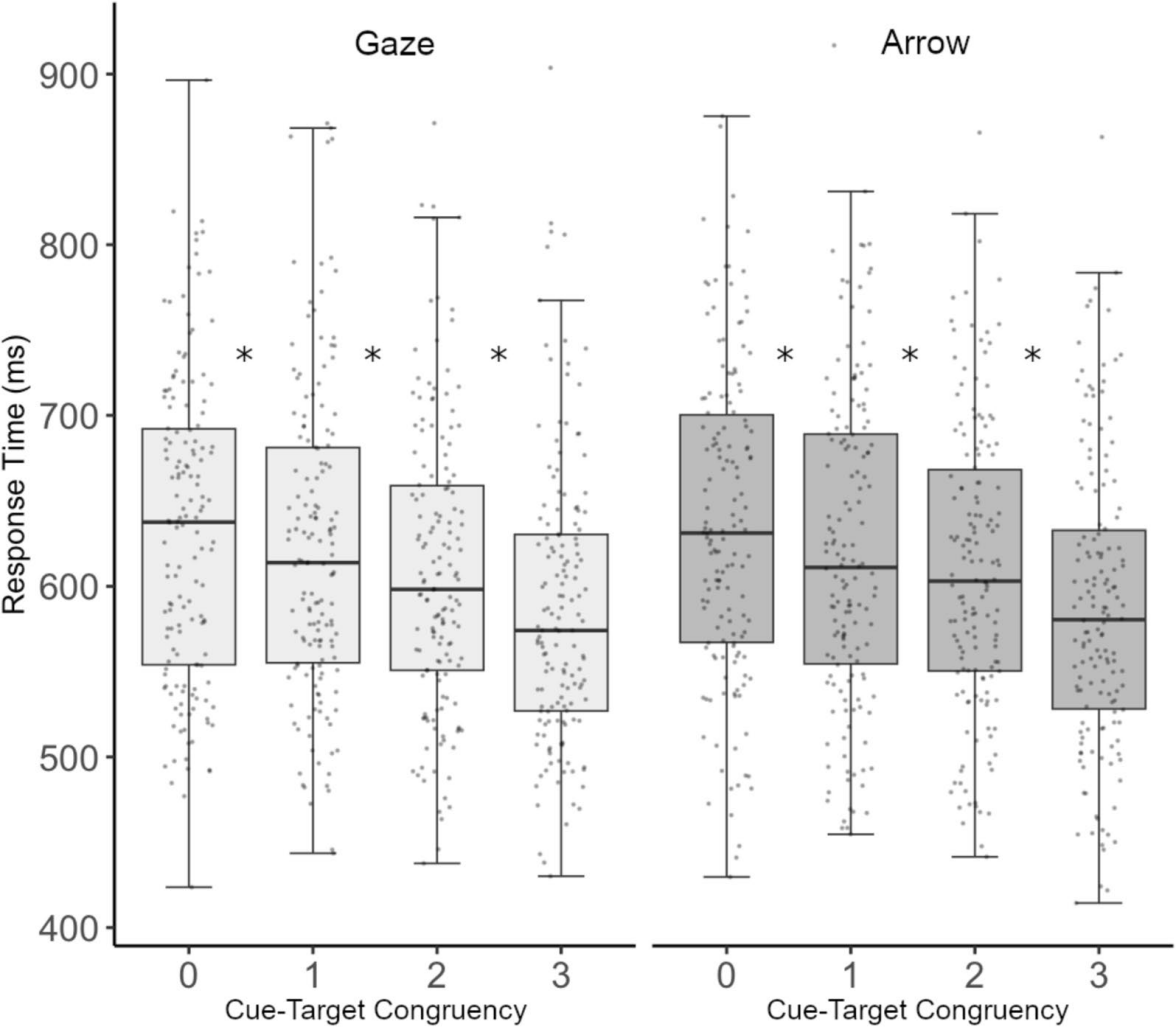
Experiment 1 Results. Box and whisker plot showing individual participants mean correct RTs as a function of Cue type and Cue-target congruency. Line within a box depicts median RT, with the box boundaries denoting the upper and lower data quartiles. Whiskers denote 1.5 IQR values. *p <.0083

Mean correct RT was examined using repeated measures Analysis of variance (ANOVA) with Cue type (gaze, arrow) and Cue-target congruency (zero, one, two, or three cues cueing the target) included as factors. Greenhouse–Geisser correction is reported when Mauchly’s test of sphericity was violated. Two-tailed paired t tests corrected for multiple comparisons using a Bonferroni method were used for post hoc comparisons. Data were collapsed across target location and target type, as they were not theoretically expected to vary across these dimensions. All analyses were conducted using JASP 0.19.3 (2024). Figure 2 visualizes the RT data as a function of Cue type and Cue-target congruency.

The ANOVA indicated a significant main effect of Cue type (*F*(1,155) = 5.457, *p* =.021, *η*^*2*^_*p*_ =.034), with faster overall RTs for targets cued by gaze cues (*M* = 631.359 ms, SE = 6.40) compared to arrow cues (*M* = 634.772 ms, SE = 6.460). A main effect of Cue-target congruency was also significant (*F*(2.458,381.041) = 193.263,* p* <.001, *η*^*2*^_*p*_ =.555), demonstrating that response times overall decreased as the number of target-congruent cues increased. Post hoc t tests (α <.0167) indicated that responses differed significantly for each Cue-target congruency level, (all ts $\le$ 8.928, all ps $<$ 001). The interaction between Cue type and Cue-target congruency was not significant (*F*(3,465) = 1.289,* p* =.278, *η*^*2*^_*p*_ =.008).

To understand these results within the context of the past literature (e.g., Capozzi et al., 2018), we conducted exploratory paired samples t tests which compared RTs at each level of Cue-target congruency for each Cue type separately (*⍺* <.0083). The results indicated that for gaze, responses were significantly facilitated by each additional target-congruent cue, i.e., by one cue looking at the target (0 vs 1, *t*(155) = 5.880, *p* <.001), by two cues looking at the target (1 vs. 2; *t*(155) = 6.364, *p* <.001), and by three cues looking at the target (2 vs. 3; *t*(155) = 5.842, *p* <.001). The same pattern of results emerged for arrows (0 vs. 1, *t*(155) = 7.590, *p* <.001; 1 vs. 2, *t*(155) = 6.627, *p* <.001, 2 vs. 3, *t*(155) = 7.860, *p* <.001).

Thus, the data indicated that while target responses were consistent with previous reports in that a minority of target-congruent cues significantly sped up performance, here, using a more powerful design, we found that responses were also significantly enhanced by each additional target-congruent cue. Overall, responses for gaze cues were faster, with no other cue specific differences.

### Discussion

Experiment 1 examined target response times in human observers viewing groups of three gaze or arrow cues with varying proportions of target-congruent cues. The data indicated faster overall responses when gaze served as cues and faster target responses when one face looked toward the target compared to zero, replicating Capozzi et al. (2018). The data also indicated significantly faster responses with each additional target-congruent cue, demonstrating an effect of increasing cue-target congruency numerosity on target responses. These numerosity increases were found across both cue types. As such, these results both replicate and extend Capozzi et al. (2018) such that initial response facilitation emerges with one target-congruent cue but continues to be facilitated with additional target-congruent cues, proceeding similarly for gaze and arrow stimuli. Experiment 2 examined whether these effects varied with group size.

## Experiment 2

To investigate if these results varied with group size, in Experiment 2, we increased the group size to five. Previous research has found that in groups of five, majority of faces looking at a target was required for target-facilitated responses (Capozzi et al., 2018). It remains unknown if similar results occur with other directional cues like arrows. To examine these questions, we used the same methodology as in Experiment 1 but presented participants with images of groups of five gaze or five arrow cues. Based on past work, we expected to find that the majority of target-congruent gaze and arrow cues would be needed to elicit facilitated responses. The study was preregistered at https://osf.io/tqky3.

## Methods

### Participants

Hundred and sixty-four new participants (women = 62%, men = 38%, average age = 24.53 ± 6.25) were recruited from the same platforms and using the same criteria as in Experiment 1 (SONA, *N* = 75, Prolific *N* = 89). Data from 154 participants were analyzed after exclusions. Informed consent was obtained from all participants, and the methods and procedures were approved by McGill University’s Ethics Board.

### Apparatus, stimuli, design, and procedure

**Fig. 3:**
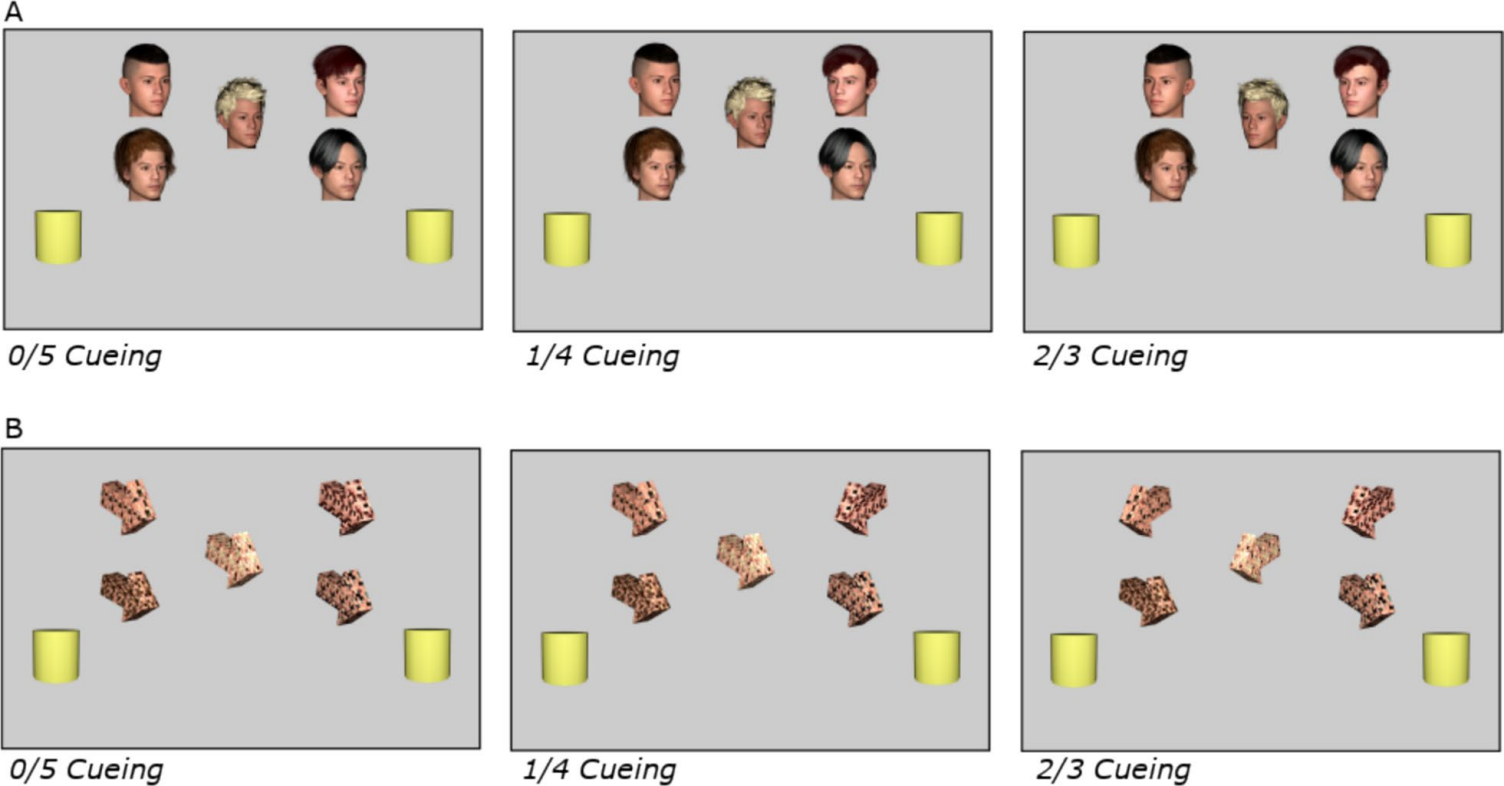
Example conditions for Experiment 2 gaze (**A**) and arrow (**B**) cues. Panels depict displays with zero or five, one or four, and two or three target-congruent cue conditions

All parameters were identical to Experiment 1, except that *(i)* stimuli included two additional identities to total five faces and five arrows and *(ii)* cue-target congruency could be zero, one, two, three, four, or five target-consistent cues as illustrated in Fig. 3.

This design had 480 trials which were presented in 6 blocks of 80. Half the trials presented gaze cues, and the other half presented arrow cues. Each Cue consistency condition was shown 80 times. The target appeared on the left and right equally often, and the target type (H or N) varied equiprobably. As before, all stimulus identities appeared in each location equally often, and no parameters were predictive of target location or target type. Ten practice trials were run at the start. The experiment took about 35 min.

## Results

**Fig. 4:**
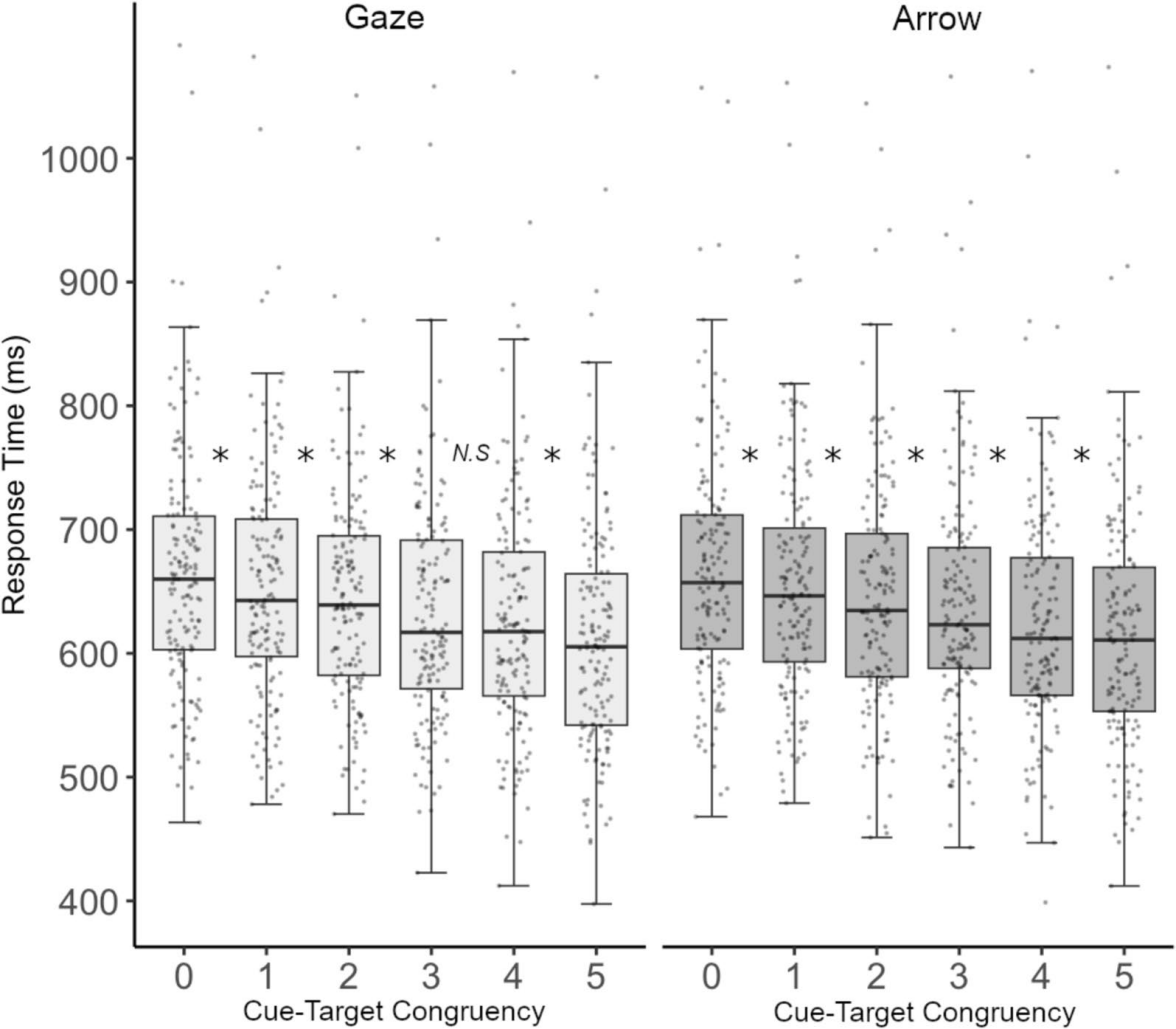
Experiment 2 Results. Box and whisker plot showing individual mean correct RTs as a function of Cue type and Cue-target congruency. Line within a box depicts median RT, with the box boundaries denoting the upper and lower data quartiles. Whiskers denote 1.5 IQR values. *p <.006

Analyses mirrored Experiment 1, with the exception that the Cue-target congruency varied from zero to five. Response accuracy was once again high at 91.86% and was not examined further. Anticipations (RT < 200 ms) and timed-out responses (RT > 1200 ms) accounted for.65% and 3.81% of trials, respectively. Inaccurate trials, trials with RT anticipations, and RT time outs were removed from data and were not analyzed further. Figure 4 visualizes the RT data as a function of Cue type and Cue-target congruency.

Based on past literature (e.g., Capozzi et al., 2018; Sun et al., 2017), we expected that the majority of target-congruent gaze cues would be needed for facilitated target responses. The repeated measures ANOVA returned only a significant main effect of Cue-target congruency (*F*(3.360,514.113) = 121.920,*p* =.001,*η*^2^_*p*_ =.443), demonstrating overall faster responses with increases in target-congruent cue numerosity. Follow-up t tests (*⍺* <.005) indicated significant facilitation between each adjacent level of Cue-target congruency (all ts $\le$ 4.313, all ps <.001). No other effects were significant (Cue type, *F*(1,153) = 1.962, *p* =.163, *η*^2^_*p*_ =.013; Cue type x Cue-target congruency, (*F*(4.881,746.772) =.430, *p* =.823, *η*^2^_*p*_ =.003).

Once again, we conducted exploratory paired samples t tests to compare RTs at each level of target congruency for each cue type separately (*⍺* <.006). The results indicated that responses were significantly facilitated by each adjacent target-congruent gaze cue (0 vs. 1, *t*(153) = 3.189, *p* =.002; 1 vs. 2 (*t*(153) = 3.118, *p* =.002; 2 vs. 3, t(153) = 4.437, *p* <.001; 4–5, *t*(153) = 4.455, *p* <.001), except for 3 vs. 4 target-congruent gaze cues, *t*(153) = 2.350, *p* =.020, which did not statistically differ. Responses differed significantly for each adjacent target-congruent arrow cue as well (all t$\text{s}\le$ 4.374, all ps <.001).

To sum, in contrast to prior work which suggested that the majority of target-congruent gaze cues were needed to elicit faster responses when groups of five served as stimuli (e.g., Capozzi et al., 2018), the present data indicated that responses for both gaze and arrow cues were initially facilitated by one target-congruent (gaze or arrow) cue as well as with incremental increases in cue-target congruency numerosity. Once again, no statistically significant differences across cue types were found.

## General discussion

In the present study, we investigated how humans responded to multiple social and non-social cues in groups of different sizes. Participants were asked to identify peripheral targets after seeing a group of three (Experiment 1, see also Supplementary Materials) or five (Experiment 2) gaze or arrow cues, which varied in their spatial congruency with respect to the target. Overall, regardless of cue type or group size, a minority of target-congruent cues (i.e., 1/3 and 1/5) was sufficient to significantly facilitate responses. Behavioral facilitation resumed with each additional target-congruent cue with these effects not varying across gaze and arrow cue types. Together, these results suggest that responses in group contexts are affected by both quorum-like evaluation thresholds and numerosity evaluation of visual cue consistency. We next discuss three points related to these findings.

First, this study was motivated by an examination of the potential use of a quorum-like evaluation in human perception. Quorum sensing, or behavior elicited by a relevant proportion of consistent group cues, reflects responding based on a threshold of consistent cues, or a quorum, which changes as a function of group size and does not always correspond to a group majority (Ward et al., 2012). Thus, quorum sensing is often marked by a nonlinear response pattern, whereby once a response threshold is met, the behavior becomes much more likely to occur, and the response function plateaus (Cornforth et al., 2014; Webster & Laland, 2008). Results from Capozzi et al. (2018) were consistent with this idea, as significant facilitation of target responses in groups of three was found with one target-congruent cue (representing a group minority) and was not facilitated further with addition of more target-congruent gaze cues (i.e., two target-congruent cues) until complete congruency (i.e., 3/3) was reached (see also Capozzi et al., 2021). The present results replicate this minority influence or the quorum-like effect, in that target responses in groups of three were facilitated by one target-congruent cue. However, here, in a more powerful design, we additionally show that increases in numerosity of target-congruent cues influenced responses as well, such that each additional adjacent target-congruent cue also significantly sped up target responses. These results extend Capozzi et al. (2018) to suggest that while responses to group gaze cues are affected by a minority threshold, increased numerosity of congruent cues can also significantly facilitate responses. It is important to note that while one could argue that these data are fully consistent with the numerosity evaluation, they are also consistent with the notion that a minority of target-congruent cues lead to facilitated target responses, which dovetails with the notion that in small groups minority proportions of relevant cues are sufficient to affect cognitive processing (Capozzi et al., 2018; for discussion, see Ristic & Capozzi, 2022). These results also parallel behavior found in the animal kingdom, such as honeybees, who also use both quorum threshold and numerosity information in behavioral decisions. Honeybees use scout bees to find a new hive location, and once a threshold number of scout bees signaling a new location is reached (i.e., quorum), the hive becomes more likely to move to a new location. The signal to move becomes more salient as additional scout bees signal the new location (i.e., numerosity; Seeley et al., 1991). This is consistent with our data showing that both quorum-like threshold and numerosity increase in cue-target congruency significantly affected speed to respond to the target. Future investigations are needed to more precisely address the individual contributions of numerosity versus quorum-like perception in group contexts of varying sizes.

Second, our results also indicated little significant differences in results between the two cue types, i.e., gaze and arrow. Based on animal work (e.g., Franks et al., 2015; Seeley et al., 1991; Ward et al., 2008, 2012), it is reasonable to expect that quorum sensing could be specific to social information which is contained in gaze. In our experiments, however, we found that changes in cue-target congruency of both social gaze and directional arrows did not produce statistically significant differences on target responses. While past research has also found similar effects of single gaze and arrow cues on social orienting (e.g., Dalmaso et al., 2020; Galfano et al., 2012; Kuhn & Kingstone, 2009; Ristic et al., 2002, 2007), it has been argued that some of those effects could be attributed to the directionality information that is shared by the two cues (Capozzi & Ristic, 2020). Our data support the idea that the directionality of cues could be at least partially responsible for the behavioral effects of cue congruency. Future studies in which social saliency within the group is increased could be of value. For example, the two types of cues (social vs. non-social) could be manipulated in opposition, or group members could display salient biological signals (e.g., emotions) or social characteristics that are connected or disconnected from the observer (e.g., in/out group ethnicity or gender).

Third, our results did not reveal differences in data for groups of three and groups of five. In groups of three, we found that a single target-congruent cue facilitated responses, with speed of responses significantly decreasing with each additional target-congruent cue. Similarly, in groups of five, performance was facilitated with one target-congruent cue and maintained the same trend of response speeding with increases in cue-target congruency numerosity. One possibility is that the group size difference between three and five in the present study was not sufficient to represent the difference between small and large group. Research on group size suggests that small groups are typically comprised of two to four individuals, while a crowd is considered as a group of ten and more (Ristic & Capozzi, 2022). Thus, groups of five fall in between this nominal distinction between small and large group and could be treated flexibly as either small or large depending on context. Indeed, research by Gallup et al. (2012) suggests that in real world crowds, about ten consistent gaze cues are needed to elicit reliable gaze following, with additional target-congruent gazers making little difference on the subsequent observers’ gaze-following behavior. Thus, while the quorum threshold response may change in larger groups, research suggests that at least for gaze cues, response facilitation driven by increasing cue numerosity may level off at about ten target-congruent cues. Future work is needed to understand if quorum threshold responding is affected when group sizes are increased beyond five in experimental tasks.

## Conclusions

In sum, here we found that responses to various levels of visual information consistency in multi-cue group settings proceed similarly when social gaze and non-social arrow serve as cues, utilizing both a quorum-like and a numerosity evaluation processes, with facilitated responses emerging in response to a minority of the cue-congruent cues as well as with additional target-congruent cues irrespective of group size. Thus, human behavior appears to be sensitive to different levels of visual spatial consistency of cues in the environment and may be facilitated flexibly via both quorum threshold and numerosity increases in environmental cue consistency.

## Supplementary Information

Additional file 1.

## Data Availability

Anonymous data from participants consenting to sharing of their data are available at https://osf.io/b3xac/ for Experiments 1 and 2 and at https://osf.io/n36tz/ for the data reported in Supplementary Materials.
