# Supplementary material for: Flexible use of quorum and numerosity principles in evaluation of social and non-social cues in group contexts
Source: Cogn Res Princ Implic. 2026 Jan 12;11:7. doi: 10.1186/s41235-025-00703-9 (PMC12791076; doi:10.1186/s41235-025-00703-9)
Supplement: Supplementary file 1 — Additional file 1. [file 41235_2025_703_MOESM1_ESM.docx]

**Supplementary Materials**

Flexible use of quorum and numerosity principles in evaluation of social and non-social cues in group contexts

Jessica Savoie^1^, Francesca Capozzi ^2^ and Jelena Ristic^1^

*^1^Department of Psychology, McGill University, Montreal, Quebec, Canada*

*^2^Department of Psychology, University of Quebec in Montreal (UQAM), Montreal, Quebec, Canada*

**Control Experiment**

Spatial arrangement could influence perceived cohesiveness of a social group, and in turn affect responses. To investigate if differences in group spatial arrangements affected the results of Experiment 1, in this control experiment, we used the same group spatial arrangement as Capozzi et al. (2018). To extend this original investigation, here we also included arrows as a comparison condition. If the effects found in Experiment 1 reported in the main text were affected by differences in group spatial formation, here we expected to find target response enhancement with one target-congruent gaze cue, without further speed enhancement with increasing cue-target congruency numerosity. If such responses were unique to gaze cues, we expected to find the pattern of responses to diverge across gaze and arrow cue types. The study was preregistered at [https://osf.io/xawy7](https://can01.safelinks.protection.outlook.com/?url=https%3A%2F%2Fosf.io%2Fxawy7&data=05%7C02%7Cjelena.ristic%40mcgill.ca%7Cbe7f13b2458546e27e6908de014645e2%7Ccd31967152e74a68afa9fcf8f89f09ea%7C0%7C0%7C638949598499167240%7CUnknown%7CTWFpbGZsb3d8eyJFbXB0eU1hcGkiOnRydWUsIlYiOiIwLjAuMDAwMCIsIlAiOiJXaW4zMiIsIkFOIjoiTWFpbCIsIldUIjoyfQ%3D%3D%7C0%7C%7C%7C&sdata=PtBwA0MT3q3NfBorNGShaSfRuxx2XRiKayje1SZbzRU%3D&reserved=0).

**Methods**

**Participants**

A group of 159 new participants (female=92%, male=.06%, other=.02%, age=20.59$\pm$2.024) were recruited using the same volunteer pool, inclusion and exclusion criteria as reported in Experiment 1. Data from 150 participants were analyzed. Informed consent was obtained from all participants, and the methods and procedures were approved by the University’s Research Ethics Board.

**Apparatus, Stimuli, Design, and Procedure**

All parameters were kept identical to Experiment 1, except that (*i*) stimuli included a yellow sphere (2cm), (*ii*), as in Capozzi et al. (2018), face and arrow cues were presented in a line formation, as shown in Figure 1SM, and (*iii*) since the cues in Capozzi et al. (2018) were faces only, the presentation of cue type was blocked, with the order of blocks randomized for presentation order across participants. Therefore, participants responded to one cue type per block of trials^[[1]](#footnote-1)^.


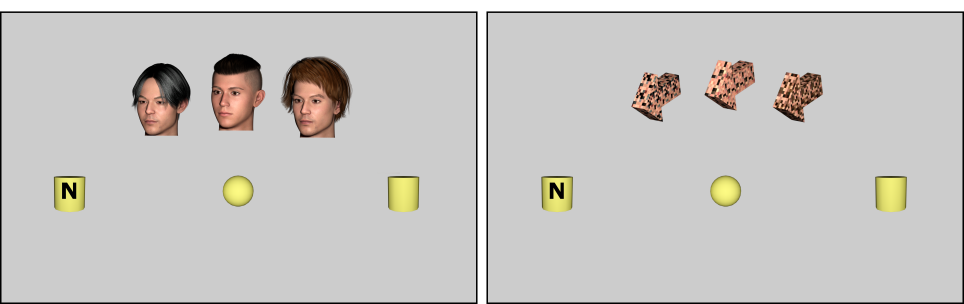


**Figure 1SM**. **Illustration of the group spatial arrangement.** Stimuli are not drawn to scale.

**Results**

Analyses and statistical parameters mirrored Experiment 1. Response accuracy was high at 91.24% and was not analyzed further. Timed out (RT>1200ms) and anticipatory responses (RT<200ms) accounted for 2.4% and .36% of trials respectively and were removed from further analyses.

If group’s spatial arrangement influenced the pattern of results in Experiment 1, a minority of target-congruent gaze cues should lead to faster responses, without further significant enhancement of responses with increases in cue-target congruency numerosity.

Figure 2SM visualizes the RT data as a function of Cue type and Cue-target congruency. The repeated measures ANOVA analyzing mean correct RTs as a function of Cue type and Cue-target congruency returned only a significant main effect of Cue-target congruency (*F*(2.6,387)=97.459, *p*<.001, *η^2^_p_*=.001; Cue type *F*(1,149)=.192, *p*=.662, *η^2^_p_*=.395; Cue type x Cue-target congruency, *F*(2.7,413.53)=.385, *p*=.749, *η^2^_p_*=.003), demonstrating a significant decrease in RTs with Cue-target congruency increases. Significantly faster (*⍺=*<.0167) responses were found for each level of Cue-target congruency (all *t*s $\leq$4.529, all *p*s<.001). Exploratory paired samples t-tests (*⍺*<.0083) indicated that responses were significantly faster for each additional target-congruent cue for each cue type (Arrow all *t*$s\leq$6.246, all *p*$s$<.002; Gaze all *t*$s\leq$6.06, all *p*$s<.$003).


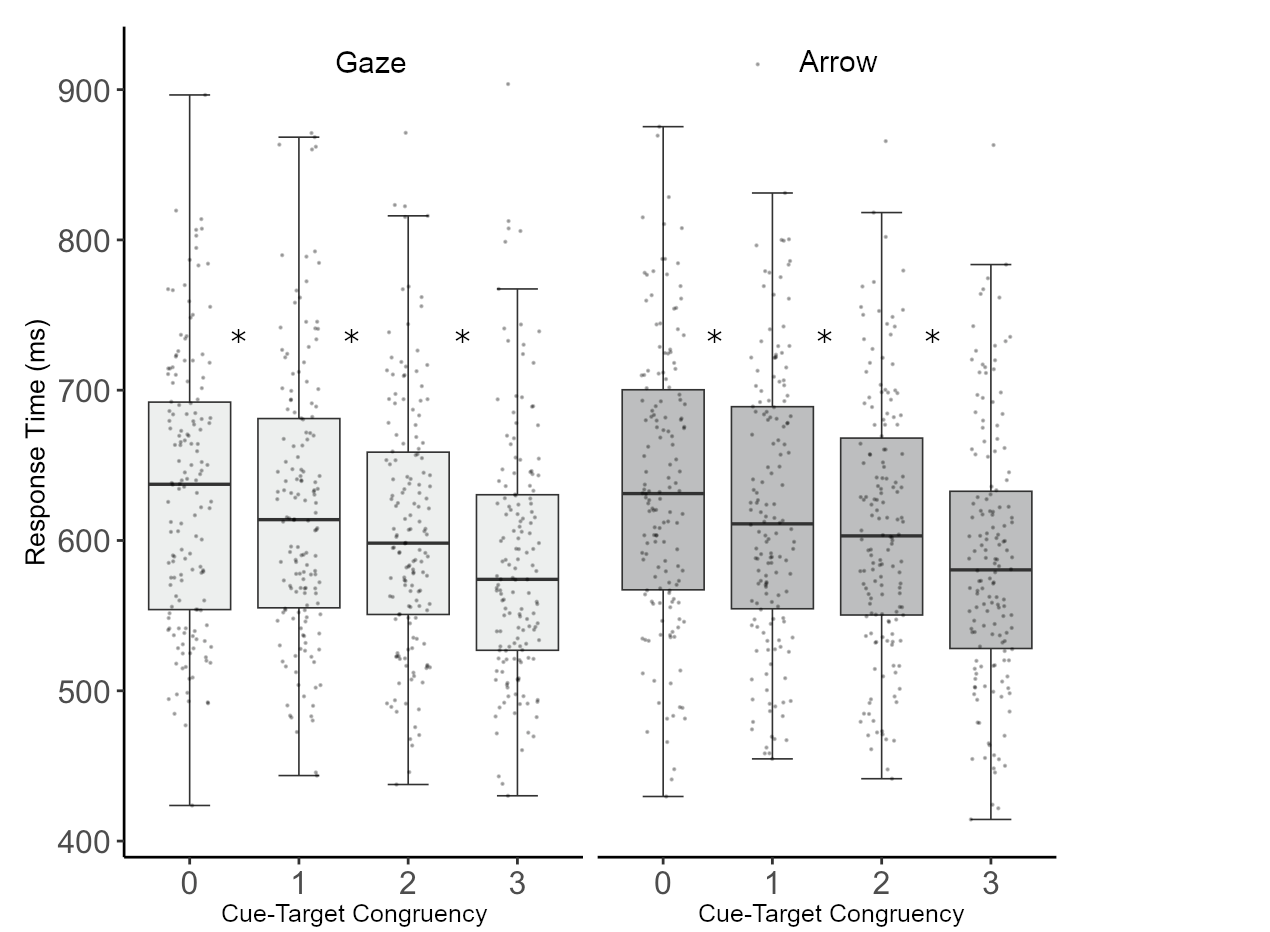


**Figure 2SM. Results.** Box and whisker plot showing individual participants mean correct RTs as a function of Cue type and Cue-target congruency. Line within a box shows group median RT, and the box boundaries represent the upper and lower data quartiles. Whiskers denote 1.5 IQR values. **p*<.0083.

**Discussion**

This control experiment used the same variables as Experiment 1 reported in the main text, except that the group members were arranged in a line-up spatial formation. The data fully replicated Experiment 1 to show that a minority of target-congruent gaze and arrow cues produced significant response enhancements, while increases in cue congruency numerosity further significantly influenced responses across both gaze and arrow cues.

1. Due to a balancing issue with the experiment programming, the first 24 trials from each cue consistency condition per cue type were analyzed rather than the first 36. [↑](#footnote-ref-1)
